# *Angiostrongylus vasorum*, *Aelurostrongylus abstrusus*, *Crenosoma vulpis* and *Troglostrongylus brevior* Infections in Native Slug Populations of Bavaria and Baden-Wuerttemberg in Germany

**DOI:** 10.3390/pathogens11070747

**Published:** 2022-06-30

**Authors:** Lisa Segeritz, Katharina Mareike Westhoff, Roland Schaper, Carlos Hermosilla, Anja Taubert

**Affiliations:** 1 Institute of Parasitology, Faculty of Veterinary Medicine, Justus Liebig University Giessen, 35392 Giessen, Germany; katharina.westhoff@vetmed.uni-giessen.de (K.M.W.); carlos.r.hermosilla@vetmed.uni-giessen.de (C.H.); anja.taubert@vetmed.uni-giessen.de (A.T.); 2 Independent Researcher, 51381 Leverkusen, Germany; roland.schaper@gmx.de

**Keywords:** metastrongyloids, gastropod-borne diseases, *Angiostrongylus vasorum*, *Aelurostrongylus abstrusus*, *Troglostrongylus brevior*, *Crenosoma vulpis*

## Abstract

*Angiostrongylus vasorum, Crenosoma vulpis*, *Aelurostrongylus abstrusus* and *Troglostrongylus brevior* can cause severe cardiovascular and pulmonary symptoms in companion animals and wildlife. Recently, these nematodes were reported to spread within Europe and South America. The reasons behind this are still unknown, but obligate gastropod intermediate host populations might play a role. Therefore, lungworm infections in terrestrial slug populations in selected geographic areas of the Federal States of Bavaria and of Baden-Wuerttemberg, Germany, were studied. In total, 517 slugs (462 *Arion* spp., 51 *Deroceras reticulatum*, one *Limax maximus*, and three unknown slug species) were collected in the summer and autumn seasons, artificially digested and microscopically and molecularly analyzed for the presence of metastrongyloid lungworm larvae. Overall, gastropods showed a prevalence of 11.61% (60/517) for *A. vasorum*, 1.74% (9/517) for *A. abstrusus*, 0.77% (4/517) for *C. vulpis* and 0.97% (5/517) for *T. brevior* infections, respectively. In Obrigheim (Baden-Wuerttemberg), a hyperendemic focus of canine angiostrongylosis was identified. Here, gastropod infection rates rose from 13.60% (17/125) to 62.96% (34/54) within a few months. In total, 25.61% (84/328) of analysed terrestrial gastropods from Baden-Wuerttemberg were positive for metastrongyloids. In contrast, Bavarian gastropods showed a much lower prevalence of 4.76% (9/189). For the first time, the presence of *T. brevior* was confirmed for *Arion* spp. in Baden-Wuerttemberg via molecular analyses. Overall, the current data confirm that canine angiostrongylosis occurs in hyperendemic foci in certain geographic areas with high infection rates in intermediate host populations. As a result, the prevalence for a specific region can rise remarkably within a short period of time. Thus, for a better understanding of lungworm epidemiology in Germany and to protect dogs from angiostrongylosis in hyperendemic foci, it seems mandatory to enhance current efforts on Metastrongyloidea-targeted monitoring on a geographical and time span-related level.

## 1. Introduction

*Aelurostronylus abstrusus*, *Angiostrongylus vasorum*, *Crenosoma vulpis* and *Troglostrongylus brevior* all belong to the superfamily Metastrongyloidea and represent a group of nematodes with a heteroxenous life cycle, infecting companion animals (dogs, cats) and mammal wildlife as definitive hosts, gastropods as intermediate hosts, and several paratenic hosts (e.g., birds, amphibians, reptiles) [1,2,3].

*A. vasorum*, also called the French heartworm, is a highly pathogenic lungworm which causes severe and occasionally fatal angiostrongylosis in canine definitive hosts, including cardiovascular, respiratory, neurological and systemic disorders. Angiostrongylosis is a gastropod-borne disease with a broad intermediate host range of terrestrial pulmonate gastropods. In Europe, red foxes (*Vulpes vulpes*), wolves (*Canis lupus*) and gold jackals (*Canis aureus*) act as reservoir hosts [4,5,6,7,8,9]. The carnivore definitive host becomes infected by uptake of third-stage larvae (L3) released from the intermediate host through the ingestion of L3-infected gastropods or infected paratenic hosts. The adult nematodes mainly reside in the *arteria pulmonalis* or the right heart of the definitive host [10].

Sharing a similar life cycle, but showing significant differences in the localization of adult nematodes and thereby also in pathogenicity, *C. vulpis*, the fox lungworm, resides in the bronchi and bronchioles of canids and potentially induces bronchitis, bronchiolitis and interstitial pneumonia [11,12].

*Troglostrongylus brevior* and *A. abstrusus* infect domestic cats and wild felid species, such as wild cats (*Felis silvestris* Schreber, 1777) and lynxes (*Lynx lynx* Linnaeus, 1758) [13,14,15,16]. Whilst *T. brevior* is rarely reported, *A. abstrusus* seems the most prevalent feline lungworm species, occurring worldwide and causing granulomatous pneumonia with sometimes severe clinical signs in domestic cats [15,17]. *T. brevior* was only reported in restricted regions and recent studies indicated that it is a rather specific parasite of European wild cats (*F. silvestris*) [18,19,20]. Nevertheless, this parasite has currently gained more attention since a considerable number of troglostrongylosis cases were reported in mainly juvenile domestic cats [21,22,23]. Recent studies indicate that *T. brevior* was probably overlooked, and in some areas, this parasite was recently diagnosed more often than *A. abstrusus* in stray cats from Jerusalem [23].

Metastrongyloid lungworms seem to spread and emerge in countries where it has previously not been reported [1,5,24,25,26]. *Angiostrongylus vasorum*, *A. abstrusus, C. vulpis* and *T. brevior* were consistently found in areas of Europe, South America, Africa and North America, where these parasites were supposed to be non-endemic [1,5,12,24,25,27,28,29,30,31]. Furthermore, *A. vasorum* infections seem to expand with a northward tendency [24,32] in Europe and South America [33]. Of note, the causes of emergence of this parasitosis remain to this point unknown [1]. One reason for that is the fact that lungworm epidemiology is multifactorial [1,28,32]. Different parameters, such as ecological and behavioral factors as well as the climate/global warming influencing the dynamics of reservoir host populations and intermediate host populations are hypothesized to contribute to the increasing prevalence of these nematodes [1,29,34,35,36]. Therefore, studies on native gastropod intermediate host populations are crucial to better understand the complex epidemiology of metastrongyloids in various biomes.

In several European countries like Italy, Switzerland, Denmark, Germany and the United Kingdom, studies on the occurrence of *A. vasorum* and other lungworms in definitive hosts were performed [4,5,21,28,35,36]. Furthermore, in Germany, a number of related studies were carried out [12,35,37,38,39]. Similarly, data on lungworm prevalence in wild foxes [39] and domestic dog populations identified several endemic foci for Germany [35].

In contrast to plenty of data on prevalence, it is clear that less reports exist on metastrongyloid parasite infections in native gastropod intermediate host populations in Europe (e.g., UK, Denmark, Germany and Spain), showing a patchy/spatial geographical distribution of *A. vasorum* slug infections [25,30,36,40,41,42].

The first data on the prevalence of *A. vasorum* and other metastrongyloid lungworm larvae in German mollusk intermediate hosts refer to selected areas of Hesse and Rhineland-Palatinate [36]. Thereby, some hotspots with high *A. vasorum* prevalence in slugs (up to 19%) were identified and seasonal differences were stated [36]. Based on the latter findings, this study complements previous data on intermediate host prevalence by filling the gap for non-studied regions in Southern Germany. Therefore, natural slug populations from selected regions of the Federal States of Bavaria and Baden-Wuerttemberg were here investigated for the presence of metastrongyloid larvae by gastropod digestion, morphometric microscopy and molecular analyses.

## 2. Results

### 2.1. Prevalence of Angiostrongylus vasorum, Aelurostrongylus abstrusus and Crenosoma vulpis in Native Terrestrial Slug Populations

In each Federal State, two main collection sites were sampled and in each sampling area, four different sites were probed in summer and autumn. Three of four investigated counties (i.e., Obrigheim, Walldürn and Bad Brückenau) were identified as positive regions for lungworm larvae-infected slugs. In Lohr am Rhein (Bavaria), positive gastropods were not detected at any time point.

Of the sampled gastropods, 17.99% (93/517) were infected by metastrongyloid larvae (Table 1). For microscopically lungworm-positive samples (*n* = 81), molecular analyses were performed to detect ITS2 sequences, which were used for BLAST-based identification to species level (Table 2). Thus, four metastrongyloid lungworm species, namely *A. vasorum*, *A. abstrusus*, *C. vulpis* and *T. brevior* were detected in this study (Table 2). Furthermore, molecular analyses diagnosed larvae to species level in some microscopically doubtful cases (5/17), where destroyed larval structures impeded morphological species identification. 

**Figure 1:**
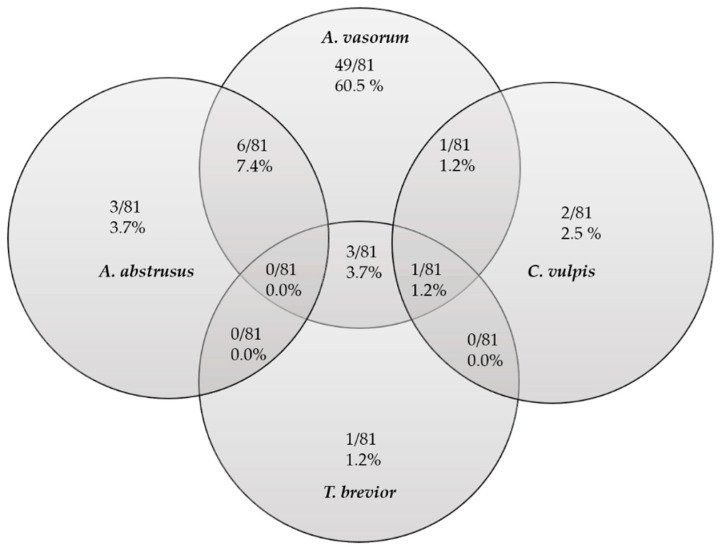
Mono-, co- and triple-metastrongyloid infections in native German slug populations with *Angiostrongylus vasorum*, *Crenosoma vulpis*, *Aelurostrongylus abstrusus* and *Troglostrongylus brevior*.

When considering gastropod infections with one or more lungworm species by combining microscopic and molecular analyses, several cases of co- (*n* = 10) and one triple-infection (*C. vulpis*, *T. brevior* and *A. vasorum*) were detected (Figure 1). There was no co-infection of *C. vulpis* and *A. abstrusus* observed. In the case of *A. vasorum*, most infected gastropods (49/60, 81.67%) showed a single species infection. 18.33% (11/60) of *A. vasorum*-infected gastropods harbored at least one other lungworm species.

The overall most frequent parasite species found was *A. vasorum*, with a total prevalence of 11.61% (60/517) (Table 1). Conversely, a lower prevalence was detected for *A. abstrusus* with 5.65% (9/517), for *T. brevior* with 0.97% (5/517) and for *C. vulpis* with 0.77% (4/517). Furthermore, 2.90% (15/517) of the investigated slugs harbored metastrongyloid larvae which could not be identified microscopically to species level due to destroyed morphological relevant structures, i.e., damage at the posterior and/or anterior extremity or cuticle.

Of 517 collected slugs, microscopic analysis revealed 81 lungworm larvae-positive samples. Those were analyzed for mono- and co-infections with the different lungworm species via microscopic and molecular analyses. Not shown in this figure are the slugs with non-identifiable metastrongyloid infections (15/81).

Interestingly, seasonal differences in gastropod lungworm prevalence were observed which seemed sampling site-dependent. Hence, an outstanding rise in *A. vasorum* prevalence from summer to autumn was detected in the county of Obrigheim in Baden-Wuerttemberg. Thus, within a few months, *A. vasorum* prevalence increased by more than 4.5-fold from 13.60% in summer to 62.96% in autumn (Table 1). At this sampling site, the prevalence of *A. abstrusus* also increased within these two seasons by more than 10-fold from 0.80 to 11.11% (Table 1). At the other sampling sites, no consistency in seasonal changes was observed.

### 2.2. Slug Species and Larval Burden

Collected terrestrial gastropods (*n* = 517) were assigned to three slug genera, i.e., *Arion, Limax* and *Deroceras*. According to species level, 89.36% (462/517) of them corresponded either to large red slugs (*Arion rufus*) or the invasive (alien) Spanish slugs (*Arion vulgaris*), followed by 9.86% (51/517) of gray garden slugs (*Deroceras reticulatum*), and 0.19% (1/517) of leopard slugs (*Limax maximus*). In 0.58% (3/517) of slug species, identification neither to species nor to genus level was possible to achieve.

**Figure 2:**
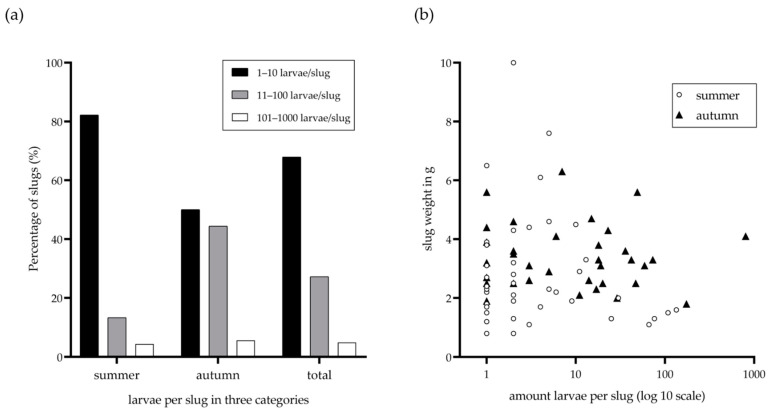
Metastrongyloid larval burdens in terrestrial slugs of Germany in summer and autumn. Graph (**a**) indicates larval burden categories for slug lungworm infections. The proportion of slugs harboring 1–10, 11–100 and 101–1000 metastrongyloid larvae per specimen is depicted. Correlation of slug weight and metastrongyloid larval burden is shown in graph (**b**) each dot represents a slug collected in summer and each triangle indicates a slug collected in the autumn season. The X axis is shown as a nonlinear logarithmic scale; the Y axis is shown as a linear scale.

Lungworm larvae-positive gastropods all were *Arion* spp. slugs (92/93), and with one exception, the only collected *L. maximus* was also harbouring *A. vasorum*-L3. In total, 67.90% (55/81) of metastrongyloid larvae-positive gastropods hosted ≤ 10 larvae, 27.20% (22/81) were infected with 11–100 larvae, and four (4.90%) showed a high larval burden of > 100, but less than 1000 larvae (Figure 2). The overall highest larval burden was observed in an *Arion* spp. slug carrying 802 larval stages, collected in Obrigheim in autumn. We furthermore observed seasonal differences in larval burden and slug weight. Accordingly, during summer, the slugs were infected with a lower larval burden than in autumn, where more gastropods were carrying more than 10 larvae/slug (Figure 2). Likewise, the mean larval burden per g slug tissue increased from 7.98/g in summer to 12.80/g in autumn. Overall, the mean slug weight was 3.00 g and the mean larval burden was 25.48 larvae/slug and 10.12 larvae/g slug tissue, respectively. 

## 3. Discussion

The current epidemiological study confirms that various common terrestrial slugs, such as *A. vulgaris*, *A. rufus, D. reticulatum* and *L. maximus* indeed act as competent intermediate hosts for metastrongyloid lungworms in southern parts of Germany [34]. Of high interest, in the county Obrigheim (Baden-Wuerttemberg), a hyperendemic focus for *A. vasorum* was identified with a prevalence of 62.96% in slug populations in autumn. In contrast, at Bavarian sampling sites, gastropod *A. vasorum* prevalence in general proved low, showing a maximum value of 0.89%, thereby confirming the well-known patchy distribution of *A. vasorum* infections in high and low endemic geographic regions. In line with this, other epidemiological studies on gastropod intermediate hosts also showed sampling site-dependent variations of lungworm prevalence ranging from 1.6% to 43% in terrestrial slugs, and were also reported on a classical patchy distribution pattern in this host type [36,40,41,42]. Likewise, *A. vasorum* prevalence in domestic and wild canid definitive hosts in Germany were also characterized by various hyperendemic foci strongly varying from 8.4% to 27.3% within different Federal States [35,39].

Furthermore, referring to the hyperendemic focus in Obrigheim, the gastropod prevalence was rising remarkably within months with the season, showing a more than four-times higher *A. vasorum* prevalence in slugs in autumn when compared to summer-related findings. Obviously, in these areas dogs are at a very high risk of acquiring angiostrongylosis when engorging these slugs. These findings reinforce the necessity of including gastropod intermediate host populations in the study and discussion on the spread and emergence of canine angiostrongylosis in Europe, which was reported in the last decades [1,5,23,34,43]. Currently, the underlying causes of the spread of canine angiostrongylosis are not fully understood, and multiple factors, which are regionally distinct and highly context-dependent, are considered to play a role [1]. Thus, environmental parameters, such as climatic factors, edaphic composition and biodiversity with area-specific gastropod populations, consisting of a diversity of species, which also vary e.g., in their life cycles, behaviour, innate immune reactions and lungworm susceptibility, are leading to regionally varying prevalences [1,40,43,44,45,46,47,48].

In the current study, the invasive Spanish slug (*A. vulgaris*) and the large red slug (*A. rufus*) were the most frequently collected gastropod species. These two species have a similar phenotype and can only be differentiated morphologically as juveniles or by dissection of the reproductive tract [49,50]. *Arion vulgaris* is currently the predominant slug in Germany and is considered as a pest species [51]. Nonetheless, a recent study indicated that this species is indeed native to Western Germany and by mistake was considered to be a neozoan species [51,52]. Due to high slug reproductive rates and beneficial climatic and environmental conditions, this species experienced a massive increase in population and therefore led to the impression of it being an invader rather than a native slug [51]. Members of the family Arionidae are considered as facultative carnivores, showing coprophagic behaviour and are carrion-feeding slugs [53,54]. All slug species investigated in the current study (*Arion* spp., *L. maximus* and *D. reticulatum*) are versatile opportunist feeders. Thus, agonistic behaviour towards conspecifics as well as other species, including cannibalism, has been reported [55,56,57]. Therefore, slug lungworm infections can occur via consumption of larvae present in both the faeces of definitive hosts and the tissue of dead slugs (intermediesis) [58,59]. Moreover, all analyzed slug species are known to be susceptible to metastrongyloid lungworm infections, as previously demonstrated [23,34,46,60]. In the only other epidemiological study in German geographic areas on lungworm infections in gastropods, *A. vulgaris* also represented the most collected species and the one with the highest lungworm infection rates [36]. 

*Arion vulgaris, A. rufus* and *D. reticulatum* in general have an annual life cycle [61], but semivoltine life cycles can occur as well [46,60]. In contrast, the multivoltine and iteroparous species *L. maximus* has a longer lifespan of approximately three years. Given that Arionidae grow constantly and lifelong, the collection of adult slugs weighing more than 6 g in summer might indicate that some slugs survived during winter by hibernation or that they were the first ones hatching in autumn of the previous year [47,55,62]. In some studies, gastropod weight and larval burden showed positive correlation [42], while in others, as well as in the current study, there was no statistically significant correlation [40]. Furthermore, the slugs, which weighed more than 6 g, showed rather low larval burdens (Figure 1). 

Considering seasonal influences, we documented that the larval burden/slug was higher in autumn than in summer, thereby increasing the risk of definitive host infection in a season-dependent manner. Reinfections of slugs over time and a higher intake capacity of older slug on faeces might represent influencing factors. Of note, the *A. vasorum*-related prevalence peak in dogs refers to the winter months [35,63]. Considering the prepatent period of four to eight weeks of this parasite, the high prevalence in gastropods in autumn may directly be linked to high prevalence in dogs in winter. Conversely, Lange et al. [36] detected the highest *A. vasorum* prevalence in German slugs in the summer season. 

Similar to other European epidemiological studies, *Arion* spp. slugs were found in greater numbers on collection sites [23,34,40,42,64]. Other slug species like *L. maximus* and *D. reticulatum* were also collected, but found in much lower abundance in current sampling areas. Plausible reasons could be their smaller size, less eye-catching colour or general lower occurrence in these geographic locations. Based on the current small sample size of slug species other than *Arion* spp., it remains speculative whether these species are less infected with lungworms or not. Of note, some studies suggest that *Arion* spp. acts as a preferred intermediate host [36,42]. 

It is worthwhile to mention that the climate conditions for the year 2018 of gastropod collection were much warmer and drier than previous year meteorological records in these regions [62]. The influence of climatic conditions, such as extreme drought, on slug populations and metastrongyloid lungworm infections has been controversially discussed [1,32,34,45,49,65]. As such, temperature-driven influences on gastropod populations could be the following. In general, terrestrial slugs respond to changing temperatures by an increase in their daily activities [66]. This phenomenon can often be observed after rainfall at daytime, when temperatures are falling quickly. However, temperature rises above 21 °C stimulate slug activity as well [66]. Obviously, the likelihood of lungworm transmission from the intermediate host to the definitive host will rise when slugs become more active and are thereby exposed to dogs or other hosts instead of staying in their natural hideouts. Thus, changes in temperature might cause an increase of slug activity during the dawn hours and eventually also lead to an enhancement in coprophagic/carrion-feeding behaviours. Moreover, higher environmental temperatures were also documented to positively influence the development of *A. vasorum* and *A. abstrusus* larvae within infected gastropods [45,48].

The current study showed that the dynamics of *A. vasorum* prevalence is fast-changing and highly fluctuating. Hence, data on lungworm prevalence have to be interpreted with care since rapid changes might be seen. Assuming that the spread of *A. vasorum* can be triggered by different factors, such as intermediate host population dynamics and optimal environmental conditions, any means of early intervention to interrupt the parasite’s life cycle in regions where it has not yet disseminated into other intermediate host populations seems beneficial to combat further spreading. However, it can be challenging to identify early lungworm infections in dogs and cats, since clinical signs vary strongly and subclinical cases are common [3,11,64,65,67]. Hence, lungworm infections are easily overseen by both owners and veterinarians. Consequently, veterinary health staff need to be aware of the importance of prophylaxis, preventive treatments and routinely performed screening tests [1,68]. Moreover, pet owners can play an important role in reducing the spread of lungworm infections by their hygiene practices in collecting dog and cat faeces, whenever possible. The fact that pet owners routinely travel with their companion animals to non-endemic areas clearly rises the risk of geographical parasite transmission. Likewise, the closely related feline neuro-angiotropic metastrongyloid parasite *Gurltia paralysans*, which was originally exclusively reported as endemic in South America [69,70], has recently been recorded in a cat in Spain, presumably due to the import of undiagnosed *G. paralysans*-infected cats to Europe [71]. 

As a take-home message, future lungworm-related studies should comprise long-term investigations and include both gastropod population-based estimations and faecal analyses on wild definitive hosts (e.g., foxes, wolves, wild cats or lynxes) in the same regions [4,13,39,72,73]. Moreover, interdisciplinary approaches are required to resolve the complex relationships between the gastropod intermediate host, intermediate host population dynamics, intermediate host species spectrum, paratenic host, climate, environment and metastrongyloid lungworm infections in domestic and wild definitive hosts.

## 4. Materials and Methods

### 4.1. Gastropod Sampling

**Figure 3:**
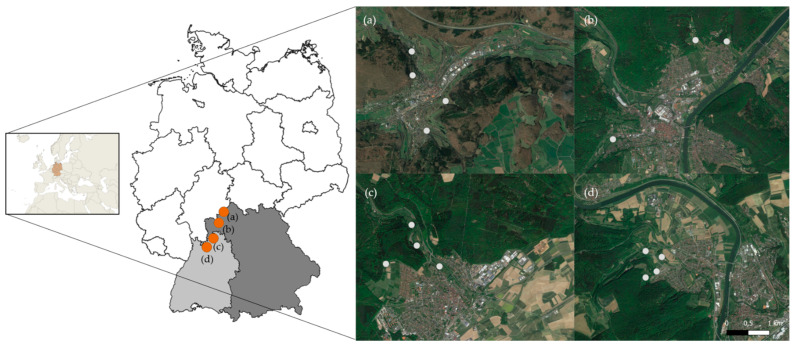
Sampling sites in Bavaria (**a**,**b**) and Baden-Wuerttemberg (**c**,**b**). (**a**) Bad Brückenau; (**b**) Lohr am Main; (**c**) Walldürn; (**d**) Obrigheim. Light grey area indicates Baden-Wuerttemberg and dark grey area Bavaria. Orange dots represent the main sampling areas. White dots indicate the four sampling locations within one area.

**Figure 4:**
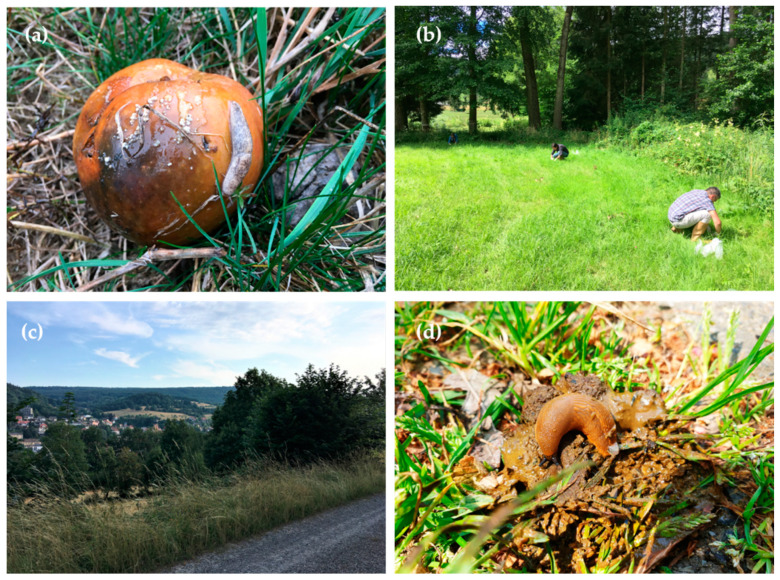
Slug species and collection environments. (**a**) *Deroceras reticulatum* feeding on an overripe apple, Lohr am Main; (**b**) gastropod collection at a meadow, Obrigheim, Baden-Wuerttemberg; (**c**) sampling site in Bad Brückenau, Bavaria; (**d**) *Arion* sp., feeding on dog faeces.

Overall, 517 slugs were analyzed, with 393 specimens being collected in the summer season and 124 in autumn of 2018. In total, 328 slugs originated from Baden-Wuerttemberg and 189 slugs originated from Bavaria. Sampling areas with reported prevalence for *A. vasorum* were chosen based on previously published data [35]. Other criteria for the selection of sampling sites included forested landscape and areas with opened grassland, the presence of water bodies such as streams, and proximity to sub-urban human settlements (potential utilization for dog walking). Within these selected locations, we assumed a high potential/probability for metastrongyloid lungworm infections, since foxes, gastropods and companion animals would share the same environment. Thus, four different main areas/cities (Figure 3 and Figure 4), two in Bavaria (Bad Brückenau: 50°18′06.6″ 9°47′24.5″ E and Lohr am Main 50°00′46.2″ N 9°35′04.3″ E) and two in Baden-Wuerttemberg (Walldürn: 49°35′24.7″ N 9°20′46″ E and Obrigheim: 49°21′01.1″ N 9°04′39.3″ E) were chosen. Within each main sampling area, three to four single collection sites were selected via GPS tracking on Google maps (https://www.google.de/maps, accessed on 8 February 2018) (Figure 3).

Since slugs are more active at dawn, early in the morning and during temperature changes, sampling was adapted to these conditions and conducted on days of forecasted rainfall, starting at 6:00 a.m. for approximately 3 h. In total, 517 slugs (393 in summer and 124 in autumn) of four different species (*A. rufus, A. vulgaris*, *D. reticulatum*, *L. maximus*) were collected by hand (Figure 4).

According to current national animal protection laws of Germany, permission for gastropod collection or their use for basic research purposes is not required.

### 4.2. Processing of Gastropod Samples

Each slug was morphologically identified based on characteristics according to [49]. Slugs were cryo-euthanized and stored at −20 °C until further investigation [41]. Gastropod samples were processed as described before [36,41] via artificial HCl/pepsin digestion. Thereafter, in order to remove any undigested material, the samples were sieved twice through a 300 µm and a 25 µm-metal sieve (Retsch, Haan, Germany) according to Segeritz et al. [25]. Remnants of the last sieving process were examined via an optical microscope (Olympus BH-2^®)^, equipped with a digital camera (SC30^®^, Olympus, Tokyo, Japan) at 40×, 200× and 400× magnification. The morphological characteristics of larvae were documented by digital photography, and larvae were counted and carefully collected by pipetting (Pasteur pipette, Hirschmann GmbH & Co. KG, Heilsbronn, Germany). The larvae were stored at 4 °C for further examination.

### 4.3. Morphological Identification of Lungworm Species

Lungworm larvae were identified by typical morphometric characteristics [72,74,75,76,77]. Therefore, body width and length, oesophagus form (non-rhabditiform), ratio of esophagus to body length (1:3–1:2) as well as tail morphology of larvae was analyzed, as reported elsewhere [22,29,34].

### 4.4. DNA-Based Confirmation of Lungworm Species

DNA from pooled metastrongyloid larvae from one slug was isolated by using a commercial kit (Quiagen DNeasy Blood and Tissue Kit^®^) and analyzed as reported previously [26,36]. Molecular analyses were performed by conventional PCRs using the nematode forward primer NC1 5′ACGTCTGGTTCAGGGTTGTT-3′ and the reverse primers NC2 5′-TTAGTTTCTTTTCCTCCGCT-3′ and MetR 5′-CCGCTAAATGATATGCTTA-3′ [73,78]. Thereafter, direct sequencing was performed by sending DNA amplicons of samples (*n* = 12) to a commercial service (LGC Genomics, Berlin, Germany). Resulting sequences were processed by the software Chromas (version 2.6.6) and consensus sequences were compared with sequences deposited in GenBank via the BLAST algorithm (http://www.ncbi.nlm.nih.gov/BLAST, accessed on 15 April 2022).

## 5. Conclusions

To the best of our knowledge, this is the first study on the prevalence of metastrongyloids in obligate intermediate hosts in southern parts of Germany, i.e., in the Federal States of Bavaria and Baden-Wuerttemberg. Thereby, the remarkable rise in the prevalence in slugs of *A. vasorum* within a period of a few months at one location demonstrated the complexity of *A. vasorum* epidemiology and confirmed the presence of hyperendemic foci in Germany. Moreover, metastrongyloid prevalences in the genus *Arion* confirmed its role as a pivotal intermediate host of canine angiostrongylosis/crenosomosis as well as feline aelurostrongylosis/troglostrongylosis in Germany. Overall, an interdisciplinary approach in future research projects is required in order to evaluate the complex relationship between gastropod populations, paratenic hosts, climate, environment and lungworm infections. Moreover, veterinary health staff and pet owners should be aware of preventive means and pursue the early treatment of lungworm infections in definitive hosts.

## Figures and Tables

**Table 1:** Lungworm prevalence in gastropods from Baden-Wuerttemberg and Bavaria, Germany.

Province	Baden-Wuerttemberg				Bavaria				All locations		
County	Walldürn		Obrigheim		Bad Brückenau		Lohr am Main				
Season	Summer	Autumn	Summer	Autumn	Summer	Autumn	Summer	Autumn	Summer	Autumn	Total
	prevalence in % (number of positive samples/numbers of analysed gastropods)										
*Angiostrongylus vasorum*	5.41% (8/148)	0.00% (0/1)	13.60% (17/125)	62.96% (34/54)	0.89% (1/112)	0.00% (0/41)	0.00% (0/8)	0.00% (0/28)	6.62% (26/393)	27.42% (34/124)	11.61% (60/517)
*Aelurostrongylus abstrusus*	0.00% (0/148)	0.00% (0/1)	0.80% (1/125)	11.11% (6/54)	0.89% (1/112)	2.44% (1/41)	0.00% (0/8)	0.00% (0/28)	0.51% (2/393)	5.65% (7/124)	1.74% (9/517)
*Crenosoma vulpis*	1.35% (2/148)	0.00% (0/1)	1.60% (2/125)	0.00% (0/54)	0.00% (0/112)	0.00% (0/41)	0.00% (0/8)	0.00% (0/28)	1.03% (4/393)	0.00% (0/124)	0.77% (4/517)
*Troglostrongylus brevior*	2.70% (4/148)	0.00% (0/1)	0.80% (1/125)	0.00% (0/54)	0.00% (0/112)	0.00% (0/41)	0.00% (0/8)	0.00% (0/28)	1.27% (5/393)	0.00% (0/124)	0.97% (5/517)
Metastrongylidaea *	4.73% (7/148)	0.00% (0/1)	0.80% (1/125)	1.85% (1/54)	5.36% (6/112)	0.00% (0/41)	0.00% (0/8)	0.00% (0/28)	3.56% (14/393)	0.81% (1/124)	2.90% (15/517)
Total	14.19% (21/148)	0.00% (0/1)	17.6% (22/125)	75.93% (41/54)	7.14% (8/112)	2.44% (1/41)	0.00% (0/8)	0.00% (0/28)	12.98% (51/393)	33.87% (42/124)	17.99% (93/517)
	25.61% (84/328)				4.76% (9/189)				17.99% (93/517)		

* Not further morphologically identified.

**Table 2:** Molecular identification of metastrongyloid larvae from native German slugs by BLAST search of their ITS2 sequences.

Location	Season	Detected Parasite	Accession Number	Homology (in %)	Identity (in %)
Obrigheim (BW ^1^)	Summer	*Troglostrongylus brevior*	OK481078	100	99.8
Obrigheim (BW)	Summer	*Crenosoma vulpis*	OK465458	100	100
Bad Brückenau (BY ^2^)	Summer	*Aelurostrongylus abstrusus*	OK481077	100	98.66
Walldürn (BW)	Summer	*Troglostrongylus brevior*	OK480968	100	100
Walldürn (BW)	Summer	*Troglostrongylus brevior*	OK480959	100	99.79
Walldürn (BW)	Summer	*Troglostrongylus brevior*	OK481081	100	100
Walldürn (BW)	Summer	*Troglostrongylus brevior*	OK480958	100	99.80
Obrigheim (BW)	Autumn	*Aelurostrongylus abstrusus*	OK481075	99	99.52
Obrigheim (BW)	Autumn	*Aelurostrongylus abstrusus*	OK480967	100	100
Obrigheim (BW)	Autumn	*Aelurostrongylus abstrusus*	OK481083	100	99.09
Obrigheim (BW)	Autumn	*Aelurostrongylus abstrusus*	OK481082	100	100
Obrigheim (BW)	Autumn	*Aelurostrongylus abstrusus*	OK481076	100	99.78

^1^ BW: Baden-Wuerttemberg; ^2^ BY: Bavaria.

## Data Availability

The sequences obtained from German slugs were deposited in GenBank database (National Center for Biotechnology Information, NIH, Bethesda, USA) (https://www.ncbi.nlm.nih.gov/genbank/) and are available under accession numbers OK481078, OK465458, OK481077, OK480968, OK480959, OK481081, OK480958, OK481075, OK480967, OK481083, OK481082 and OK481076.
